# Effect of cement type and thickness on push-out bond strength of fiber posts

**DOI:** 10.15171/joddd.2018.043

**Published:** 2018-12-19

**Authors:** Farzaneh Farid, Khadijeh Rostami, Sareh Habibzadeh, MohammadJavad Kharazifard

**Affiliations:** ^1^Department of Prosthodontics, School of Dentistry, Tehran University of Medical Sciences, Tehran, Iran; ^2^DDS, School of Dentistry, International Branch, Tehran University of Medical Sciences, Tehran, Iran; ^3^Department of Prosthodontics, School of Dentistry, International Branch, Tehran University of Medical Sciences, Tehran, Iran; ^4^Statistics Advisor, Dental Research Center, Tehran University of Medical Sciences, Tehran, Iran

**Keywords:** Dental Adhesives, dental dowel, resin cement

## Abstract

***Background.*** This study investigated the effect of the thickness of two resin cements on push-out bond strength of fiber posts to root dentin.

***Methods.*** Sixty decoronated single-rooted teeth were endodontically treated. The specimens were then randomly allocated to two groups (n=30); group 1: drill size 90 w, matching the size of fiber posts used; group 2: drill size 110, larger than the posts. The specimens in each group were divided into 2 subgroups (n=15); subgroup SE, in which the posts were cemented with self-etch Panavia F2.0 and subgroup SA, in which self-adhesive Panavia cement was used. After 72 hours, 2 slices with 1 mm of thickness were prepared from the mid-root of each specimen. Push-out bond strength test was performed in a universal testing machine at a crosshead speed of 1 mm/min. Data were analyzed with two-way ANOVA and t-test.

***Results.*** The mean bond strengths of the posts cemented in matched-size spaces for SE and SA groups were 4.02±1.6 and 4.12±2.3 MPa, respectively. For posts cemented in oversized spaces, the values were 4.9±2.3 and 2.8±1.3 MPa, respectively. In matched-size spaces, there was no significant difference between the two groups.

***Conclusion.*** The results of this study suggested that increasing the cement thickness would reduce the bond strength of fiber posts to root dentin when self-adhesive cements are used; however, self-etch adhesives were not affected.

## Introduction

Endodontically treated teeth with insufficient coronal structure are restored by post and cores, followed by crowns.^1,2^ The retention of post is important for the success and longevity of post-core-supported restorations.^3^ Based on the method of fabrication, two main types of intracanal posts are available: custom-made and prefabricated.^4^ Custom-made posts are intimately adapted to the canal walls, leaving a thin uniform cement layer.^5^ On the other hand, prefabricated posts exhibit less adaptation, particularly in oval-shaped canals.^6^ Their retention relies more on factors such as post length, diameter, shape, surface configuration and the luting agent.^7,8^ As a result, resin cements are a better choice for this post cementation.^5^ However, most of the time, the cement thickness around the post is not uniform and the greater the cement thickness, the more would be the microporosities and the polymerization shrinkage.^1,9^ This increase in microporosities and polymerization shrinkage will reduce the bond strength of the post to canal walls due to an increase in C-factor ‒ the ratio between bonded and non-bonded surfaces.^10-12^ However, Perdigao et al^13^ suggested that stress relaxation provided by the air in the structure of cement might compensate the deleterious effect of high C-factor.

Studies investigating the effect of cement thickness on the retention of posts have yielded conflicting results. Some have shown a positive effect;^5,14^ yet, others have found no effects^1,13,15^ or a reduction in bond strength.^9,16^ Some have even reported mixed results.^3,17-19^ It seems the effect of an oversized post space on the bond strength of the post to root dentin mostly depends on the type of the adhesive system and thickness and type of the luting cement.^3,9,17^ Chan et al^5^ reported that metal Paraposts cemented into loose-fitting canals exhibited greater resistance to pull-out dislodgement than posts cemented into well-fitting canals. The cements used in their study were zinc phosphate, zinc polycarboxylate, glass-ionomer and resin cement with an average thickness of 250 µm. Hagge et al^14^ reported the same results for Panavia 21 OP with 105‒125 µm of thickness. On the other hand, Schmage et al^16^ showed significant reduction in bond strength after increasing the thickness of five different resin cements, including Rely X Unicem. All the above-mentioned studies have used the pull-out test. Ozcan et al^9^ reported somewhat the same results for etch-and-rinse and self-adhesive cements after performing the push-out test, except that the thickness of self-etch cement did not significantly affect the bond strength.^9^ Perdigao^13^ and Perez et al^15^ did not report any significant effects on push-out bond strength of quartz fiber posts cemented with etch-and-rinse adhesive and self-cured Hi-X cements in oversized post spaces.

This study evaluated the effect of the thickness of two self-etch and self-adhesive resin cements on push-out bond strength of fiber posts to root dentin. The null hypothesis was that the cement thickness has no effect on bond strength.

## Methods

Sixty single-rooted teeth with 16‒18 mm of root length and straight canals, without aberrant canal morphology or size as confirmed by radiography, were included in this study. The exclusion criteria consisted of large carious lesions, root cracks or resorption, and former root canal therapy. The selected teeth were cleaned and disinfected in 0.5% chloramine T solution (Parsian Pakhsh Arna, Gilan, Iran) and stored in normal saline solution (NSS) for later use within 3 months. All the teeth were decoronated 1 mm coronal to CEJ with a diamond disc (Kerr Dental Corporation, West Collins Orange, CA United States) under water irrigation. The canals were cleaned by using K-files (Many, Inc., Tochigi, Japan) #15‒30 and shaped by K-files #35-60 using the standard step-back technique. Working length was set at 1 mm short of the actual canal length and verified for each specimen by radiography; 5.25% sodium hypochlorite (NaOCl) solution (CERKAMED-CHLORAXID, CERKAMED Dental-Medical Company, Poland) was used for irrigation between files and at the end of cleaning and shaping. All the root canals were irrigated with NSS and dried with absorbent paper points (Sinadent, Iran). Obturation was performed using standard lateral condensation technique with gutta-percha (Orca; Tiagin, China) and AH26 sealer (Dentsply, De Trey GmbH, Konstanz, Germany). The root canal orifices were covered with a eugenol-free temporary dressing (Coltosol; Coltene/Waledent, Altstatten, Switzerland). The specimens were kept at 100% humidity at room temperature. After seven days, 12 mm of gutta-percha were removed from the coronal aspect of each root canal using #1‒3 Peeso Reamers (Mani; Tochigi, Japan). The specimens were allocated to 2 groups according to the size of post space prepared. In the first half, the preparation was performed by using orange (#70) and red (#90 w) drills, and in the other half with orange, red and blue (#110) drills.

The drills used were provided by the manufacturer of the fiber posts used (Innopost drills; Innotech, Rimini, Italy). Both canals and posts were cleaned by ethyl alcohol (Ethanol, 96%) and then the canals were irrigated by NSS and dried with paper points. Each group was divided into two subgroups; SE and SA, based on the kind of cement used (n=15). In the SE subgroup, the fiber posts were cemented with self-etch cement (Panavia F2.0, Kurary Noritake Dental Inc. Okayama, Japan), and in the SA subgroup self-adhesive cement (Panavia SA cement Plus, Kurary Noritake Dental Inc. Okayama, Japan) was used. The cementation process was preformed according to the manufacturer’s instructions. The cement thickness difference between the two groups was 100 µm.

For cementing with Panavia F2.0, equal amounts of ED primer (A&B) were mixed and applied to the canals. After 30 seconds, the excess of primer was removed with paper point. Then equal amounts of A&B pastes were mixed and the posts were coated with cement and immediately inserted in the canals. The excess of cement was removed and the orifice of the root canal was light-cured for 20 seconds. Then the coronal portion of the teeth was covered by Oxyguard II (Panavia F2.0, Kuraray Noritake Dental Inc. Okayama, Japan). The cemented posts were kept under finger pressure for 7 minutes. After removing Oxygaurd, the specimens were stored at 100% humidity at room temperature for 24 hours.

For cementing with Panavia SA, equal amounts of A&B pastes were mixed for 10 seconds, applied over the posts and inserted into the canals immediately with vibrating motion. The excess cement was removed. The coronal portion of the canals was light-cured for 10 seconds. The specimens were kept under finger pressure for 7 minutes and then stored at room temperature and 100% humidity for 24 hours.

**Figure 1:**
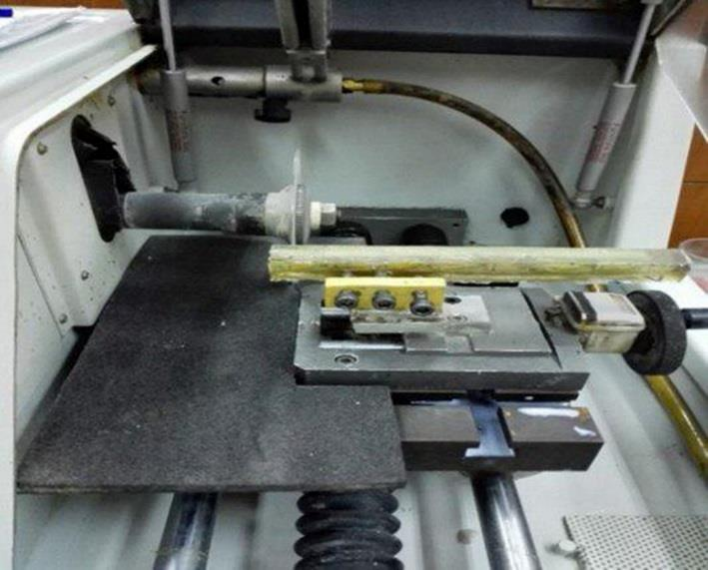
Cutting the specimens with Mecatome.

**Figure 2:**
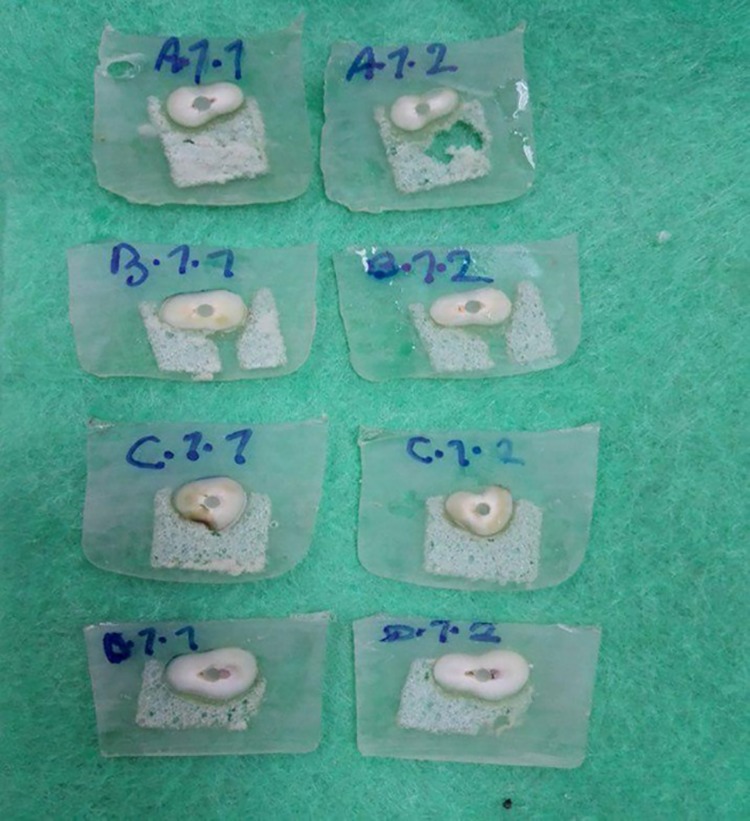
Slices of 1mm thick from mid root of each specimen.

In order to place the specimens in a precision micro-cutting machine (Mecatome T 201 A; PERSI, France), they were mounted in acrylic resin. First, they were placed on a piece of foam (Foam Tehran, Tehran, Iran). The foam was placed within a stainless steel mold and the mold was filled with cold-curing acrylic resin (Pekatray, Baye, Leverkuser, Germany). After setting, the resin block was separated from the metal mold and placed in the cutting machine. Two 1-mm-thick slices were selected from the mid-root of each specimen for the push-out test (Figures 1 and 2).

A digital caliper (Guanglu Measuring Instrument Co., Ltd, Guilin, China) was used to verify the thickness of the slices with 0.01-mm accuracy. The coronal side of each slice was marked with a pen. Under ×2 magnification survey, sections with uneven cement thickness or voids were excluded.

**Figure 3:**
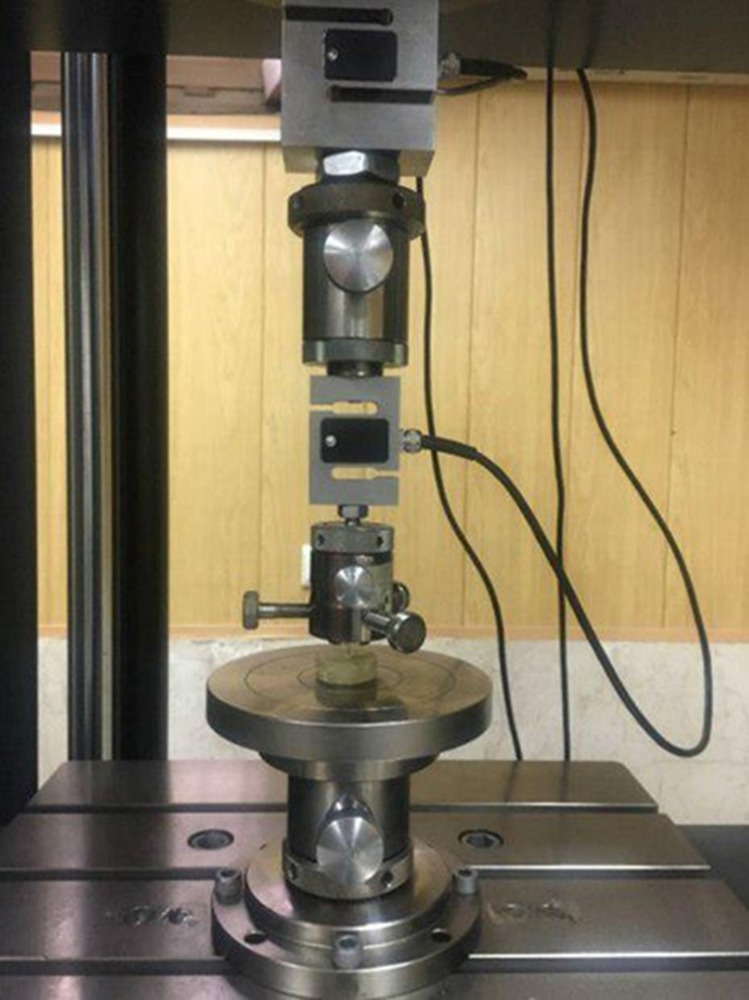
Push-out bond strength test performed by Universal Testing Machine

Push-out test was performed using a universal testing machine (Zwick-Roel, Germany). Care was taken to center the push-out pin (with a diameter of 1.0 mm) on the post surface without causing stress on the post space walls. Load was then applied to the apical side of the root slice at a crosshead speed of 1 mm/min (Figure 3). The peak force that caused extrusion of the post segment from the slice was taken as the point of bond failure and the value was recorded in Newton (N). The push-out bond strength in MPa was calculated by the following formula:

Push-out bond strength = $\frac{F}{A}=\frac{\max imum\;loaded\;force}{JI(R1+R2)\sqrt{{\left(R1-R2\right)}^{2}+h^{2}}}$

R1: radius of the post at coronal side of the specimen

R2: radius of the post at apical side of the specimen

h: the height of the slice

Failure mode was determined under a stereomicroscope (Kyky, Maillefer, China). Failures were classified as follows: (1) adhesive, between the post and resin cement; (2) adhesive, between the resin cement and root dentin; (3) mixed, with resin cement partially covering the post surface; (4) cohesive, within the fiber post; and (5) cohesive, within the dentin. Data were analyzed with two-way ANOVA and t-test.

## Results

The mean bond strengths of the groups are presented in Table 1. In matched-size spaces, no significant difference was found between the two subgroups. The mean bond strength of Panavia SA was significantly lower in oversized root canals and lower than Panavia F2.0. Increased cement thickness had no effect on Panavia F2.0. The failure modes of the specimens are presented in Table 2.

**Table 1:** Mean bond strength values in the study groups

**Post space**		**N**	**Minimum**	**Maximum**	**Mean**	**SD**
**Matched**	**Self-adhesive**	28	0.44	9.34	4.1266	2.32987
	**Self-etch**	28	1.81	7.07	4.0240	1.61162
**Oversized**	**Self-adhesive**	28	1.12	6.37	2.8532	1.34445
	**Self-etch**	28	1.46	8.92	4.9577	2.35299

**Table 2:** Mode of failure of specimens

**Drill size and cement type**	**Cohesive**	**Adhesive cement with fiber post**	**Adhesive cement with root dentin**
**matched SE**	-	27%	73%
**oversized SE**	-	14%	86%
**matched SA**	-	26%	74%
**oversized SA**	-	20%	80%

## Discussion

Resin cements are used for cementation of fiber posts because they have greater strength than other cements. However, dentin needs to be conditioned prior to the application of these cements, a time-consuming and multiple-step technique.^20^ To simplify the procedure, self-adhesive cements were introduced to dentistry.^21^ Panavia has two cement forms: the conventional self-etch cement, which is used after application of ED primer, and the self-adhesive Panavia, which does not need any conditioning of the dentin prior to cementation. This study compared the effect of cement thickness in these two cements.

The results showed that a 100-µm increase in the cement thickness had no effect on bond strength of Panavia F2.0 but significantly decreased the bond strength of Panavia SA. Therefore, the null hypothesis was rejected. Özcan et al^9^ reported the same results for Panavia F2.0 and Clearfil SA. However, significant reduction of bond strength for Clearfil SA occurred in larger cement space. Schmage et al,^16^ too, reported a decrease in bond strength in Rely X Unicem. Sahafi et al^19^ reported a moderate decrease in bond strength of Panavia F2.0 with increased cement thickness.

Most of the studies reporting no effect of cement thickness on bond strength of resin cements have used the total-etch (etch-and-rinse) technique.^1,6,13,15^ Etching dentinal walls removes the smear layer; as a result, a stronger bond is expected to be formed between dentin and the cement.^22-24^ This increase in bond strength might resist the increase in polymerization shrinkage and therefore the stress. Chan et al^5^ and Hagge et al^14^ reported an increase in bond strength of Panavia EX and Panavia 21, using the pull-out test. The difference between the results might be explained by the type of test used, and the etch and rinse technique for preparation of canals in these studies, which strengthens the bond to dentin.

There are also studies in which an increase in bond strength has been followed by a decrease after a slight increase in cement thickness.^3,17,18^ A viscous cement does not flow freely and while cementing a post into a narrow canal, most of the cement remains out of the root canal.^25^ Therefore, a slight widening of the post space helps a more favorable cement transfer into the canal. In these cases, pretreatment of the post to increase its bond to the cement could also be beneficial.

The bond strength values reported by Prado et al^17^ and Marcos et al^26^ are higher than what is reported in this study. They applied silane on their posts before cementing with a self-adhesive cement; yet, no surface treatment was performed in the current study.

The analysis of failure modes in this study revealed that most of the failures occurred at dentin‒luting cement interface. Similar results were reported by Özcan et al,^9^ Prado et al^17^ and Marcos et al.^26^ As reported, "debonding" is the most common failure in fiber-reinforced composite type of posts. Adhesion between resin cements and root dentin is difficult because of polymerization stress occurring at the cement‒dentin interface, which might be affected by root canal geometry, responsible for its high configuration factor (C-factor).^27^ The thickness of cements had no effect on the failure mode.

In this study the uniform thickness of the cement was most important; therefore, we preformed our tests on the mid-root slices of teeth, where the width of post space is mainly determined with the diameter of the drill. The apical thirds of the roots were not used because this part of the canal mainly remains untouched in routine post space preparation. The coronal third was not used either, as the canals are funnel-shaped and the drills might not completely match the canal width at this area.

The present study was carried out on human single-rooted teeth and despite all the steps taken to find identical samples, variations in the morphology of the canals were inevitable; therefore, the results showed a large standard deviation.

The push-out test, despite its limitations, was used in this study for measuring the bond strength at dentin‒cement‒post interfaces.^28^ The other possibilities would be the pull-out (tensile) and the microtensile tests. Because of the premature failure of specimens reported by Goracci et al,^29^ the microtensile test was not used.

## Conclusion

Within the limitations of this study, a 100-µm increase in post space did not affect the bond strength of fiber posts cemented with self-etch resin cement, but it significantly reduced the bond strength of posts cemented with self-adhesive cement. In post spaces, matching the post size, there was no significant difference between the bond strength of self-adhesive and self-etch resin cements. In oversized post spaces, the bond strength of self-adhesive cement was significantly lower than self-etch cement.

## Acknowledgments

This work was based on a thesis submitted to School of Dentistry, Tehran University of Medical Sciences, International Branch, in partial fulfillment of a doctorate degree in dentistry.

## Authors’ Contributions

FF and SH contributed to the concept and the design of the study and drafted the manuscript. FF supervised the project, proposed the idea, hypothesis and the experimental design. KR carried out the experiments, prepared the samples and performed the tests under supervision by FF. SH provided consultation on the whole parts of the project and proofread the manuscript. MK performed statistical analyses. All the authors have contributed to the critical revision of the manuscript and have read and approved the final paper.

## Funding

The funding for this study was partially provided by Tehran University of Medical Sciences.

## Competing Interests

The authors declare no competing interests with regards to the authorship and/or publication of this article.

## Ethics Approval

The study protocol was approved by the ethics committee of Tehran University of Medical Sciences.
